# The Gut as a Source of Infection for Fungal Pathogens: Increased Fecal *Candida albicans* Precedes Onset of *Candida* Late-Onset Sepsis in Very Preterm Infants

**DOI:** 10.1093/infdis/jiaf524

**Published:** 2025-10-10

**Authors:** Rimke R de Kroon, Irini A M Kreulen, Mark Davids, Isabelle A M van Thiel, Iris Admiraal, Xanthe Verdoes, Mirjam M van Weissenbruch, Hendrik J Niemarkt, Wouter J de Jonge, Tim de Meij, the generation P study group, Chris H P van den Akker, Aranka J van Wesemael, Nina M Frerichs, Marlou M A Kouwenberg-Raets, Christian V Hulzebos, Willem P de Boode, Marijn J Vermeulen, Daniel C Vijlbrief, Veerle Cossey

**Affiliations:** Amsterdam Reproduction and Development Research Institute, Amsterdam UMC, Amsterdam, the Netherlands; Amsterdam Gastroenterology Endocrinology Metabolism Research Institute, Amsterdam UMC, Amsterdam, the Netherlands; Department of Pediatric Gastroenterology, Emma Children's Hospital, Amsterdam UMC, Amsterdam, the Netherlands; Amsterdam Gastroenterology Endocrinology Metabolism Research Institute, Amsterdam UMC, Amsterdam, the Netherlands; Tytgat Institute for Liver and Intestinal Research, Amsterdam UMC, University of Amsterdam, Amsterdam, the Netherlands; Microbiota Center Amsterdam, Amsterdam, the Netherlands; Department of Experimental Vascular Medicine, Amsterdam UMC, Amsterdam, the Netherlands; Amsterdam Reproduction and Development Research Institute, Amsterdam UMC, Amsterdam, the Netherlands; Amsterdam Gastroenterology Endocrinology Metabolism Research Institute, Amsterdam UMC, Amsterdam, the Netherlands; Tytgat Institute for Liver and Intestinal Research, Amsterdam UMC, University of Amsterdam, Amsterdam, the Netherlands; Department of Pediatric Surgery, Emma Children's Hospital, Amsterdam UMC, Amsterdam, the Netherlands; Tytgat Institute for Liver and Intestinal Research, Amsterdam UMC, University of Amsterdam, Amsterdam, the Netherlands; Microbiota Center Amsterdam, Amsterdam, the Netherlands; Microbiota Center Amsterdam, Amsterdam, the Netherlands; Department of Experimental Vascular Medicine, Amsterdam UMC, Amsterdam, the Netherlands; Amsterdam Reproduction and Development Research Institute, Amsterdam UMC, Amsterdam, the Netherlands; Amsterdam Gastroenterology Endocrinology Metabolism Research Institute, Amsterdam UMC, Amsterdam, the Netherlands; Neonatal Intensive Care Unit, Emma Children's Hospital, Amsterdam UMC, Amsterdam, the Netherlands; Neonatal Intensive Care Unit, Maxima Medical Center, Veldhoven, the Netherlands; Department of Electrical Engineering, Technical University Eindhoven, Eindhoven, the Netherlands; Amsterdam Gastroenterology Endocrinology Metabolism Research Institute, Amsterdam UMC, Amsterdam, the Netherlands; Tytgat Institute for Liver and Intestinal Research, Amsterdam UMC, University of Amsterdam, Amsterdam, the Netherlands; Department of General, Visceral, Thoracic and Vascular Surgery, University Hospital Bonn, Bonn, Germany; Amsterdam Reproduction and Development Research Institute, Amsterdam UMC, Amsterdam, the Netherlands; Amsterdam Gastroenterology Endocrinology Metabolism Research Institute, Amsterdam UMC, Amsterdam, the Netherlands; Department of Pediatric Gastroenterology, Emma Children's Hospital, Amsterdam UMC, Amsterdam, the Netherlands

**Keywords:** *Candida*, candidemia, fungi, microbial meta-barcoding, preterm colonization

## Abstract

**Background:**

The skin-to-blood route is traditionally considered the main pathway in *Candida* late-onset sepsis (LOS) development in preterm infants. However, emerging evidence suggests that the gut also serves as a source of infection. We aimed to characterize fecal mycobiota and microbiota profiles preceding onset of *Candida* LOS to assess the role of the preterm gut microbiome in disease development.

**Methods:**

This multicenter case-control study included very preterm infants (<30 weeks of gestation) with *Candida* LOS. Each case was matched to nonaffected controls by gestational and postnatal age, hospital site, and/or cumulative antibiotic exposure prior to day of LOS onset (t = 0). Fecal samples collected at t = 0 and the 5 preceding days were analyzed by ITS1 and 16S RNA sequencing. Microbial amplicon yields, composition, and interkingdom correlations were assessed.

**Results:**

Of 2397 screened infants, fecal samples were available for 8 of 19 infants with *Candida* LOS. In these 8 cases, the ITS/16S amplicon yield ratio was increased (*P* < .001), and the relative abundance of fecal *Candida albicans* correlated positively with fungal amplicon yield (ρ = 0.71, adjusted *P* = .005), suggesting increased absolute abundance up to 5 days before onset. Additionally, bacterial yields were significantly lower (*P* = .02) and α-diversity significantly decreased (*P* = .012) when compared to the controls.

**Conclusions:**

Increased fecal *C albicans* preceded *Candida* LOS onset, implicating the preterm gut as a potential source of infection. Reduced bacterial yields and diversity suggest ecological alterations that may facilitate *Candida* pathogenicity in the preterm gut. These findings support further research into gut-derived *Candida* LOS and the potential for microbiota-targeted prevention strategies.

## Graphical Abstract

**Graphical Abstract:**
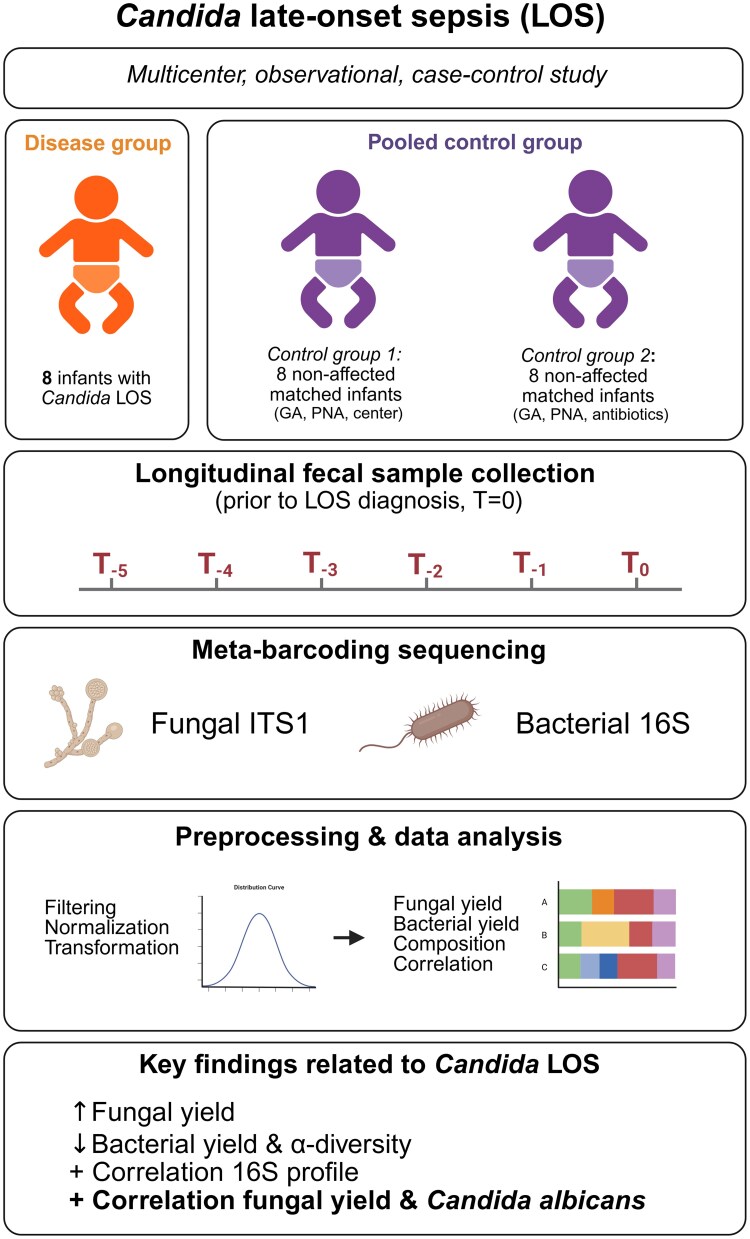
Created in BioRender. Amsterdamumc, Eminds. (2025) https://BioRender.com/w5ajcb0

## Teaser

Increased fecal *Candida albicans* preceded *Candida* late-onset sepsis in preterm infants, implicating the gut as a potential source of infection. Reduced bacterial yields and diversity suggest ecological alterations that may facilitate *Candida* pathogenicity, supporting the potential for microbiota-targeted prevention strategies.

Late-onset sepsis (LOS) remains one of the leading causes of morbidity and mortality among preterm infants [1]. Most LOS episodes in preterm infants are caused by bacterial pathogens, with a smaller proportion due to fungi, predominantly *Candida* [2–4]. The contribution of fungal pathogens varies geographically, ranging from 2% to 28% [5]. The majority of fungal LOS episodes are caused by *Candida albicans* and *Candida parapsilosis*, although other *Candida* species are emerging, such as *Candida glabrata*, *Candida tropicalis*, *Pichia kudriavzevii* (formerly *Candida krusei*), and *Candida auris*. Due to their growing resistance to antifungal agents, these species pose a serious threat to preterm infants admitted to the neonatal intensive care unit [6–8].

*Candida* LOS often follows a fulminant disease course with systemic dissemination, multiorgan failure, and death [5, 9]. Mortality rates up to 40% have been reported in preterm infants with *Candida* LOS, with survivors facing a significant risk of neurodevelopmental impairment [5, 10, 11]. However, accurate diagnosis of *Candida* LOS remains challenging because the clinical presentation is often nonspecific [12, 13]. For suspected LOS, broad-spectrum antibiotics are generally initiated to cover the most common bacterial pathogens. However, antimycotic treatment is not routinely included in empiric therapy for suspected LOS, which may delay targeted treatment for *Candida-*associated LOS. Additionally, the gold standard for diagnosis is a positive blood and/or cerebrospinal fluid (CSF) culture, which has limited sensitivity [14] and a long turnaround time [15, 16]. A deeper understanding of the underlying pathogenesis of *Candida* LOS is required to support the development of timely and accurate diagnostic strategies.

Adequate management of skin integrity in preterm infants is important as skin-to-blood transmission is considered the main pathway in the development of *Candida* LOS. However, the gut may also serve as a source for fungal pathogens, enabling their translocation across the immature intestinal mucosal barrier into the bloodstream. A similar route of infection has been proposed in the development of bacterial LOS in preterm infants, characterized by profound microbiota alterations prior to clinical onset [17–20]. However, the majority of studies testing this gut-derived hypothesis for fungal sepsis have focused on adults [21–23], with few studies conducted in the preterm population [22, 24]. The role of the preterm gut as a source of infection for *Candida* LOS remains underexplored. Therefore, the objective of this multicenter, observational, case-control study was to investigate the role of the intestinal fungal mycobiota and bacterial microbiota on the development of *Candida* LOS in very preterm infants. Elucidation of the role of the gut microbiome in *Candida* LOS pathogenesis may enable improved clinical risk stratification and identification of therapeutic targets to prevent disease progression.

## METHODS

### Cohort Design, Case Selection, and Matching Procedures

This study is embedded within a prospective, multicenter, cohort study (protocol 2014.386, amendment A2016.363). The study collects daily fecal samples and clinical data in the first 29 days of life from preterm infants (gestational age [GA], 24–30 weeks until March 2021; GA, 24–28 weeks from March 2021 onward) in 10 neonatal intensive care units in the Netherlands and Belgium. The overarching aim of this cohort study is the development of novel noninvasive biomarkers for early prediction of LOS and/or necrotizing enterocolitis (NEC). Exclusion criteria are chromosomal abnormalities and/or congenital gastrointestinal (GI) diseases. The study is approved by the local medical ethical review boards. Written informed consent was obtained from both parents or legal guardians.

**Figure 1.**
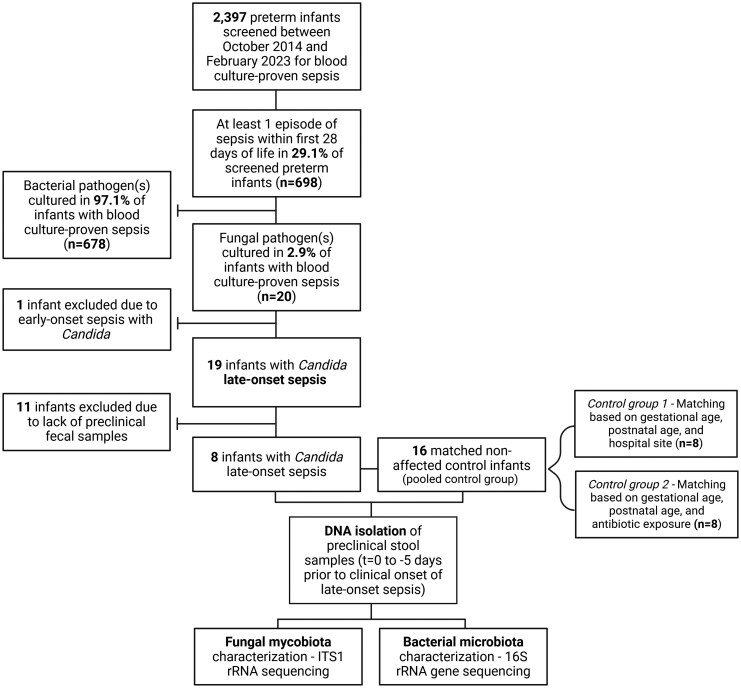
Flowchart of the infant inclusion and matching process. Mycobiota and microbiota profiles in the fecal samples of 8 preterm infants with *Candida* late-onset sepsis and 16 matched control infants were determined by ITS1 and 16S ribosomal RNA (rRNA) sequencing. Overall 2397 preterm infants were screened for culture-proven sepsis in the first 29 days of life. Infants were born in 1 of 10 neonatal intensive care units in the Netherlands and Belgium between October 2014 and February 2023. Of these, 29.1% experienced at least 1 episode of sepsis; among the infants with sepsis, bacterial pathogens were cultured in 97.1% while fungal pathogens were cultured in 2.9%. The fecal samples of 8 infants with *Candida* late-onset sepsis were available for microbiome analysis. All affected infants (n = 8) were matched to nonaffected control infants (n = 16). The pooled control group consists of 2 controls per case. Matching for control group 1 was based on gestational age (±5 days), postnatal age at diagnostic workup (t = 0) of the matched infant in the disease group, and hospital site. Matching for control group 2 was based on gestational age (±5 days), postnatal age at t = 0, and cumulative days of antibiotic administration at t = −1 (±7 days). Fecal samples were selected on t = 0 and in 5 days prior to the diagnostic workup (t = −1 to −5) for the disease and control groups. Fecal mycobiota and microbiota were characterized with ITS1 amplicon and 16S rRNA gene sequencing, respectively. Created in BioRender. Amsterdamumc, Eminds (2025) https://BioRender.com/0cz78gm

For this case-control study, all preterm infants born between October 2014 and February 2023 with a fungal LOS episode, defined as a blood and/or CSF culture–proven fungal infection, were eligible (Figure 1). Coinfections with bacterial pathogens were not excluded. Positive fungal cultures were considered contaminants and excluded from the analysis if antifungal treatment was not initiated or was discontinued after the culture results became available. For a subset of LOS episodes, fecal samples were available for microbiota and mycobiota analysis. All stool samples collected on the day of the workup that resulted in a LOS diagnosis (t = 0) and in the 5 preceding days (t = −1 to t = −5) were included for microbiome analysis.

Each LOS case was matched to 2 non-affected controls, together composing the pooled control group. All infants included between October 2014 and February 2023 were screened for the relevant matching criteria. Cases were first matched to control infants (control group 1) based on GA (±5 days), postnatal age (PNA) at t = 0, and hospital site. Controls were matched on PNA at the index day (t = 0) to ensure that fecal sampling windows were aligned. If t = 0 of the case occurred on PNA day 10, samples from the case and matched controls were collected and analyzed from postnatal days 5 through 10. Antibiotic exposure prior to onset was considered a potential confounder, given its impact on the microbiota, its association with fungal colonization and overgrowth, and its persistent effects on mycobiota diversity and *Candida* abundance [25–28 ]. To account for this, a second control infant (control group 2) was additionally matched on cumulative antibiotic exposure prior to t = 0 (±7 days) in addition to GA and PNA at t = 0. This extended matching strategy enables sub-analysis within the control groups to assess the impact of antibiotic exposure on microbial signatures. This approach minimized the potential confounding effect of rapid microbiome changes during the first month of life.

Exclusion criteria for the non-affected control infants were culture-proven early-onset sepsis (EOS) and LOS, NEC, and spontaneous intestinal perforation. EOS and LOS were defined according to the Vermont-Oxford criteria as a blood and/or CSF culture–proven infection, occurring within and after the first 72 hours of life, respectively [2]. NEC was defined according to the modified Bell's criteria as stage ≥2A [29].

### Sample and Data Collection

Fecal samples were collected in a sterile container and stored at −20 °C for short-term storage and −80 °C for long-term storage. Clinical data were collected as follows: GA, birth weight, biological sex, singleton or multiple pregnancy, Apgar scores, mode of delivery, center of birth, maternal antenatal corticosteroids, surfactant administration, mortality during NICU admittance, intravenously administered antimicrobial medication and enteral probiotics (ProPrems), feeding practices (PNA and feeding type when reaching full enteral feeding and formula administration in first 29 days of life), and a detailed description of health events. Feeding type was defined as exclusively human mother's milk (HMM), >50% HMM with human donor milk (HDM), >50% HDM with HMM, and exclusively formula feeding.

### ITS1 Amplicon and 16S Ribosomal RNA Gene Sequencing

Fecal DNA isolation, sequencing of ITS fungal and 16S bacterial profiles, and preprocessing of sequencing data are explained in detail in Supplementary Appendix A. In total, 22 fecal samples were analyzed in the disease group (n = 8 infants) and 56 samples in the pooled control group (n = 16 infants). Following the exclusion of samples with insufficient sequencing reads, the final sample counts used for 16S and ITS analysis are provided in Supplementary Table 1.

### Data Analysis, Statistics, and Software

Clinical data were analyzed in SPSS Statistics version 24 (IBM). When deemed appropriate, the independent *t* test, non-parametric test, or χ^2^ test was used. *P* ≤ .05 was considered significant.

For microbial data analysis, infants with *Candida* LOS were compared with control infants for each outcome, with analysis performed in R (version 4.3.2) and visualization in ggplot2 (version 3.5.1). To assess the impact of cumulative antibiotic exposure prior to onset on each outcome of interest, control groups 1 and 2 were first compared with each other, as well as to the disease group. When no significant differences between control groups 1 and 2 were observed for the outcome of interest, indicating a limited effect of antibiotic exposure in this setup, the individual control groups were combined into a pooled control group. This approach allowed for increased statistical power while considering the possible effect of antibiotics on the microbiota and mycobiota profiles.

To assess relative fungal and bacterial loads, polymerase chain reaction (PCR) amplicon yields (nanograms per microliter), obtained with ITS and 16S primers, were used as proxies. For each sample, input fecal DNA was normalized to ensure equal DNA input across PCR reactions (20 ng for 16S, 100 ng for ITS). This normalization minimized variation due to differences in DNA extraction concentrations. In addition, the ITS/16S amplicon yield ratio was calculated on the sample level to reduce PCR bias from unclassified, nontarget DNA fractions.

Taxonomic analyses were based on all reads, with both 16S and ITS datasets rarefied to 5000 reads before statistical testing. Two α-diversity metrics (Shannon diversity index and Faith phylogenetic diversity) were determined by using phyloseq (version 1.46.0) and picante (version 1.8.2). Differences in α-diversity or abundances of selected taxa for individual time points were assessed by the Kruskal-Wallis test. Linear mixed models were used to assess the α-diversity metrics and differential abundance of selected fungal taxa and covariate interactions, with participant identification included as a random effect to account for repeated measures and unequal numbers of samples per infant. β-Diversity was assessed by principal coordinate analysis (PCoA) based on Bray-Curtis dissimilarities. Due to the sparsity of the data, 1 sample per participant was randomly selected to test differences in β-diversity using permutational multivariate analysis of variance (PERMANOVA). To account for potential selection bias, the random sample selection was repeated 100 times, and average statistical metrics and *P* values were calculated. Correlation analysis was conducted to assess associations between fungal amplicon yield and relative abundance of selected fungal taxa by means of Spearman correlation. Correlation analysis between the microbiota and mycobiota was assessed by using the Procrustes test.

## RESULTS

### Baseline Characteristics

In total, 2397 preterm infants were screened for inclusion (Figure 1). In 20 of 698 infants (2.9%) with at least 1 culture-proven sepsis episode, a *Candida* species was the causative pathogen. Characteristics of the affected infants are displayed in Supplementary Tables 2 and 3. In 84%, *C albicans* was the causative pathogen, followed by *C tropicalis*, *C guillermondii*, and *C parapsilosis* (all 5%). Fecal samples for subsequent microbiome analysis were available for 8 infants with *Candida* LOS (Table 1). No differences in baseline characteristics were observed between this subgroup and the total cohort (Supplementary Table 2). When compared with the matched pooled control group (n = 16), affected infants were more frequently born through vaginal delivery (*P* = .046), achieved full enteral feeding later in life (*P* = .040), were less likely to achieve full enteral feeding in the first month of life (*P* = .037), and were administered more antimicrobial agents (*P* < .001; Table 1).

**Table 1.** Baseline Characteristics of the Disease Group Analyzed by ITS1 Amplicon and 16S Ribosomal RNA Sequencing and the Matched Pooled Control Group (n = 16), Consisting of Control Group 1 and 2

	Median [IQR] or No. (%)						
	Disease Group (n = 8)	Pooled Control Group (n = 16)	Control Group 1 (n = 8)	Control Group 2 (n = 8)	*P* Value^a^	*P* Value^b^	*P* Value^c^
**Demographic characteristics**							
Gestational age, wk + d (d)	24 + 5 [13]	25 + 2 [10]	25 + 3 [9]	25 + 2 [13]	.461	.527	.526
Birth weight, g	742 [279]	723 [206]	702 [196]	750 [345]	.417	.344	.636
Biological sex, female	3 (38)	8 (50)	5 (63)	3 (38)	.562	.317	>.99
Singleton	5 (63)	11 (68)	6 (75)	5 (63)	.665	.590	.549
Apgar score at 5 min	8 [4]	8 [8]	8 [2]	6 [4]	.895	.518	.343
Modus partus: vaginal delivery	8 (100)	10 (63)	5 (63.5)	5 (63.5)	.**046**	.055	.055
Maternal antenatal corticosteroids					.496	>.99	>.99
No treatment	2 (25)	2 (12.5)	0 (0)	2 (25)			
Incomplete	3 (37.5)	4 (25)	1 (12.5)	3 (37.5)			
Complete	3 (37.5)	10 (62.5)	7 (87.5)	3 (37.5)			
Mortality within NICU admittance	1 (12.5)	0 (100)	0 (0)	0 (0)	.149	.302	.302
**Medication practices**							
Surfactant treatment within 72 h					.122	.051	.141
Multiple dosages	4 (50)	6 (37.5)	1 (12.5)	5 (50)			
1 dosage	0 (0)	6 (37.5)	4 (50)	2 (25)			
No treatment	4 (50)	4 (25)	3 (37.5)	1 (12.5)			
Ratio cumulative IV antimicrobials in first 28 d of life	0.78 [0.26]	0.27 [0.34]	0.12 [0.16]	0.45 [0.21]	**<**.**001**	.**001**	.**001**
Cumulative days of IV antimicrobials at t = −1, d	11 [11]	6 [10]	4 [3]	13 [7]	.**084**	.**006**	.833
Exposure to IV antimicrobials prior to t = 0	8 (100)	16 (100)	8 (100)	8 (100)	…	…	…
Exposure to probiotics prior to t = 0	3 (37.5)	11 (31)	3 (37.5)	2 (25)	.759	>.99	.590
**Average feeding practice**							
Reached FEF within first 28 d	6 (75)	16 (100)	8 (100)	8 (100)	.**037**	.131	.131
Postnatal age at first day of FEF, d	13 [12]	10 [3]	9 [2]	12 [5]	.**040**	.**005**	.362
Feeding type at day of FEF					.608	.413	.369
Exclusively HMH	6 (100)	12 (75)	5 (62.5)	7 (87.5)			
>50% HMM + HDM	0 (0)	2 (12.5)	1 (12.5)	1 (12.5)			
>50% HDM + HMM	0 (0)	1 (6.25)	1 (12.5)	0 (0)			
Exclusively FM	0 (0)	1 (6.25)	1 (12.5)	0 (0)			
Received FEF in first month of life	2 (25)	5 (31)	2 (25)	3 (37.5)	.751	>.99	.590
Reached FEF at t = 0	3 (38)	12 (75)	7 (87.5)	5 (62.5)	.074	.**039**	.317

Each affected infant in the disease group (n = 8) was matched to 2 non-affected controls (n = 16). Infants in control group 1 (n = 8) were matched by gestational age (±5 days), postnatal age at diagnostic workup (t = 0) of the matched infant in the disease group, and hospital site. Infants in control group 2 (n = 8) were matched by gestational age (±5 days), postnatal age at t = 0, and cumulative days of antibiotic administration at t = −1 (±7 days). *P* ≤ .05 was considered significant (bold).

Abbreviations: FEF, full enteral feeding; FM, formula milk; HDM, human donor’s milk; HMM, human mother’s milk; IV, intravenous; NICU, neonatal intensive care unit.

^a^Disease group vs pooled control group.

^b^Disease group vs control group 1.

^c^Disease group vs control group 2.

### Increased Intestinal Fungal Yields and Decreased Bacterial Yields Prior to Onset of *Candida* LOS

**Figure 2.**
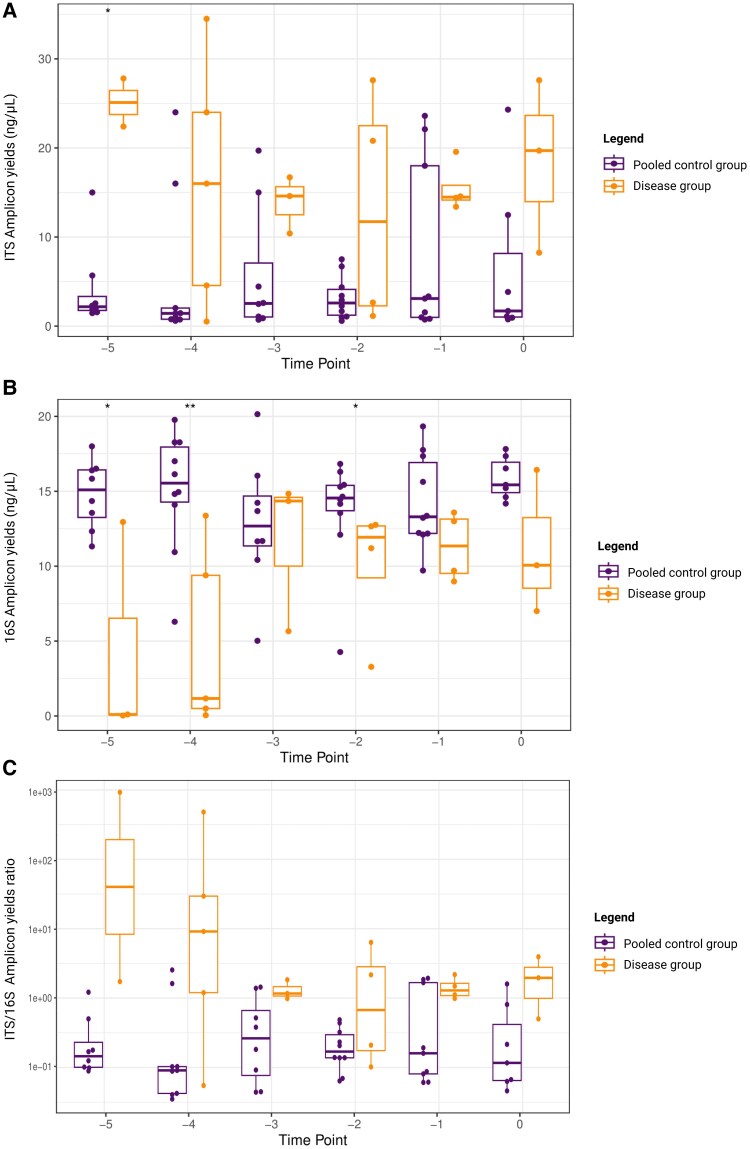
Higher fecal fungal yields, lower bacterial yields, and increased ITS/16S amplicon yield ratio in preterm infants with *Candida* late-onset sepsis as compared with the pooled control group. *A*, Fecal fungal amplicon yields determined from 5 days before diagnosis (t = −5) up to the day of diagnosis (t = 0) in infants with *Candida* late-onset sepsis (disease group, n = 8 infants; orange) vs the pooled control group (n = 16 infants; purple) (mean ± SD, fungal yield, 16.23 ± 9.43 ng/µL in the disease group vs 5.45 ± 7.17 ng/µL in the pooled control group; *P* < .001). *B*, Similarly, the fecal bacterial amplicon yields from t = −5 to t = 0 in the disease group vs the pooled control group are displayed (mean ± SD, bacterial yield, 8.68 ± 5.53 ng/µL in the disease group vs 14.39 ± 3.32 ng/µL in the pooled control group; *P* = .02). *C*, Last, the ITS/16S amplicon yield ratio over the same time interval is displayed in the disease group vs the pooled control group. The disease group demonstrated an approximately 15-fold higher yield ratio (*P* < .001). Differences in overall fungal and bacterial yields between groups over time were assessed via a linear mixed model analysis (Satterthwaite method). Statistical significance was defined as *P* ≤ .05. *P ≤ .05. **P ≤ .001. The pooled control group consisted of 2 matched controls per case. Data are presented as median (line), interquartile range (box), and minimum/maximum (error bars). Created in BioRender. Amsterdamumc, Eminds (2025) https://BioRender.com/8r23uw6

We assessed the fecal fungal and bacterial yields in the disease group and non-affected control group. No significant differences in fungal and bacterial yields were observed between the control subgroups (Supplementary Figure 1); therefore, subsequent analyses were conducted using the pooled control group. When compared to the pooled control group, the disease group showed a significantly lower bacterial yield (mean ± SD, bacterial yield, 8.68 ± 5.53 vs 14.39 ± 3.32 ng/µL; *P* = .02; Figure 2*B*) and a significantly higher fungal yield (mean ± SD, fungal yield, 16.23 ± 9.43 vs 5.45 ± 7.17 ng/µL, *P* < .001; Figure 2*A*). The disease group had an overall 15-fold higher ITS yield relative to the 16S yield from the same sample (*P* < .001), with no significant effects for the day prior to sepsis (Figure 2*C*, Supplementary Table 4). These findings indicate an overall increased absolute abundance of fecal fungal DNA and a reduced abundance of bacterial DNA prior to the onset of *Candida* LOS.

### *Candida albicans* Is the Predominant Fungal Species in Preterm Fecal Samples Before the Onset of *Candida* LOS

**Figure 3.**
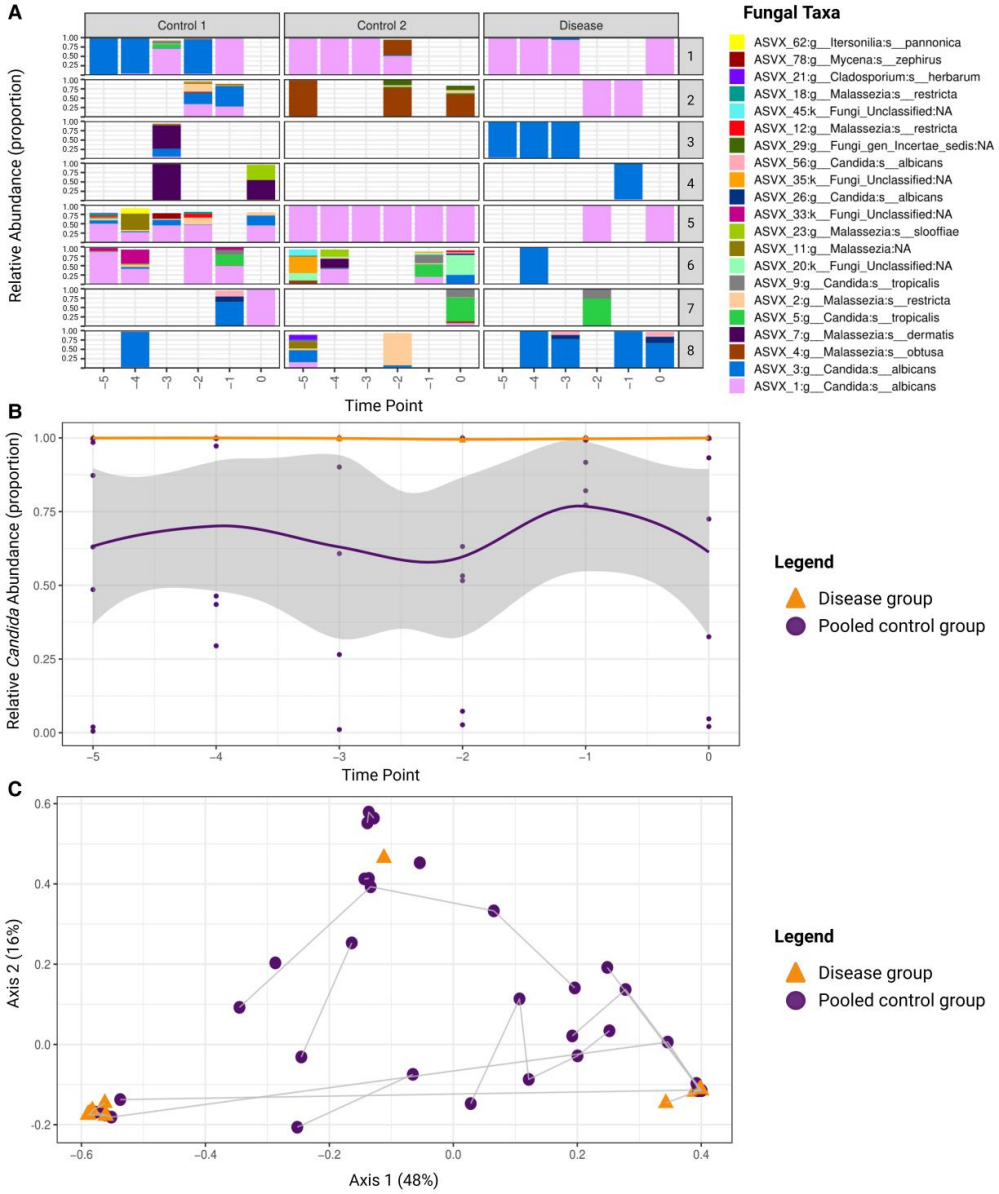
Fecal fungal mycobiota composition in preterm infants with *Candida* late-onset sepsis as compared with the pooled control group. *A*, Fungal composition plot of the relative abundance of amplicon sequence variants (ASVs) in the disease and pooled control groups. On the left y-axis, the relative abundance (proportion) of the fungal ASVs at each time point is displayed, with proportions shown at 0, 0.25, 0.50, 0.75, and 1.00. The x-axis shows the time points relative to onset of disease (t = −5 to t = 0). On the right y-axis, the 8 match groups are displayed. Each match group consists of 1 case and 2 nonaffected controls. Each color corresponds to a distinct ASV. (*B*) Taxon dynamics of the *Candida* abundance from 5 days before clinical onset (t = −5) to the day of diagnostic workup (t = 0) in infants with *Candida* late-onset sepsis (disease group, orange) vs the control infants (pooled control group, purple). The y-axis represents the RA (proportion) of *Candida*, while the x-axis indicates time (days). Differences in course of RA between the groups were assessed via linear mixed model analysis (Satterthwaite method; 0.998 ± 0.003 in the disease group vs 0.673 ± 0.368 in the pooled control group; *P* = .036). Shaded areas indicate 95% CI. Statistical significance was defined as *P* ≤ .05. *C*, Fungal β-diversity at the genus level was visualized by principal coordinate analysis based on Bray-Curtis dissimilarity (*R*² = 6.9% *P* = .27). Each point represents a single representative fecal sample per infant, colored by disease state (orange, disease group; purple, pooled control group). The gray lines connect samples belonging to the same matched group. Due to the sparsity of the data, 1 sample per participant was randomly selected to test differences in β-diversity by permutational multivariate analysis of variance. To account for potential selection bias, the random sample selection was repeated 100 times, and average statistical metrics and *P* values were calculated. Statistical significance was defined as *P* ≤ .05. Created in BioRender. Amsterdamumc, Eminds (2025) https://BioRender.com/nhykqie

To further investigate intestinal fungal signatures related to *Candida* LOS, we compared the fungal composition of the disease group vs the pooled control group, as no relevant differences in fungal signatures were observed between control groups 1 and 2 (Figure 3*A*). At the genus level, *Candida* was the predominant genus detected at all time points in the disease group, whereas the pooled control group exhibited greater variability in composition of fungal genera (Supplementary Figures 2A and 3). Despite this variability, *Candida* remained the predominant genus in the pooled control group, followed by *Malassezia* (Supplementary Figure 2A). At species level, *C albicans* was the most abundant species in both groups (Figure 3*A*). However, in all fecal samples from infants who developed *C albicans* LOS, *C albicans* was the only fungal species detected, in contrast to the control infants, who exhibited a broader variety of fungal species. Similarly, *C tropicalis* was the exclusively detected fungal species in the single available fecal sample from the infant who developed *C tropicalis* LOS.

We next assessed *Candida* relative abundance over time prior to LOS onset, which was significantly higher in the disease group as compared to the pooled control group (0.998 ± 0.003 vs 0.673 ± 0.368, *P* = .036; Figure 3*B*). A pattern of persistently high *Candida* abundance across all time points was observed in the disease group. In contrast, the pooled control group displayed greater temporal variability in *Candida* abundance. Fungal β-diversity relating to disease status at the species level was assessed by the Bray-Curtis dissimilarity index based on 1 representative fecal sample per infant. Ordination plots suggest visual heterogeneity among *Candida* sequence variants, with 1 *C. tropicalis* and 2 *C. albicans* variants observed; however, no statistically significant association on the variant level was detected, as assessed by PERMANOVA (*R*² = 6.9%, *P* = .27; Figure 3*C*), although the signal appeared to generalize at the genus level (Figure 3*B*).

### Reduced Bacterial α-Diversity in Preterm Fecal Samples Before the Onset of *Candida* LOS

**Figure 4.**
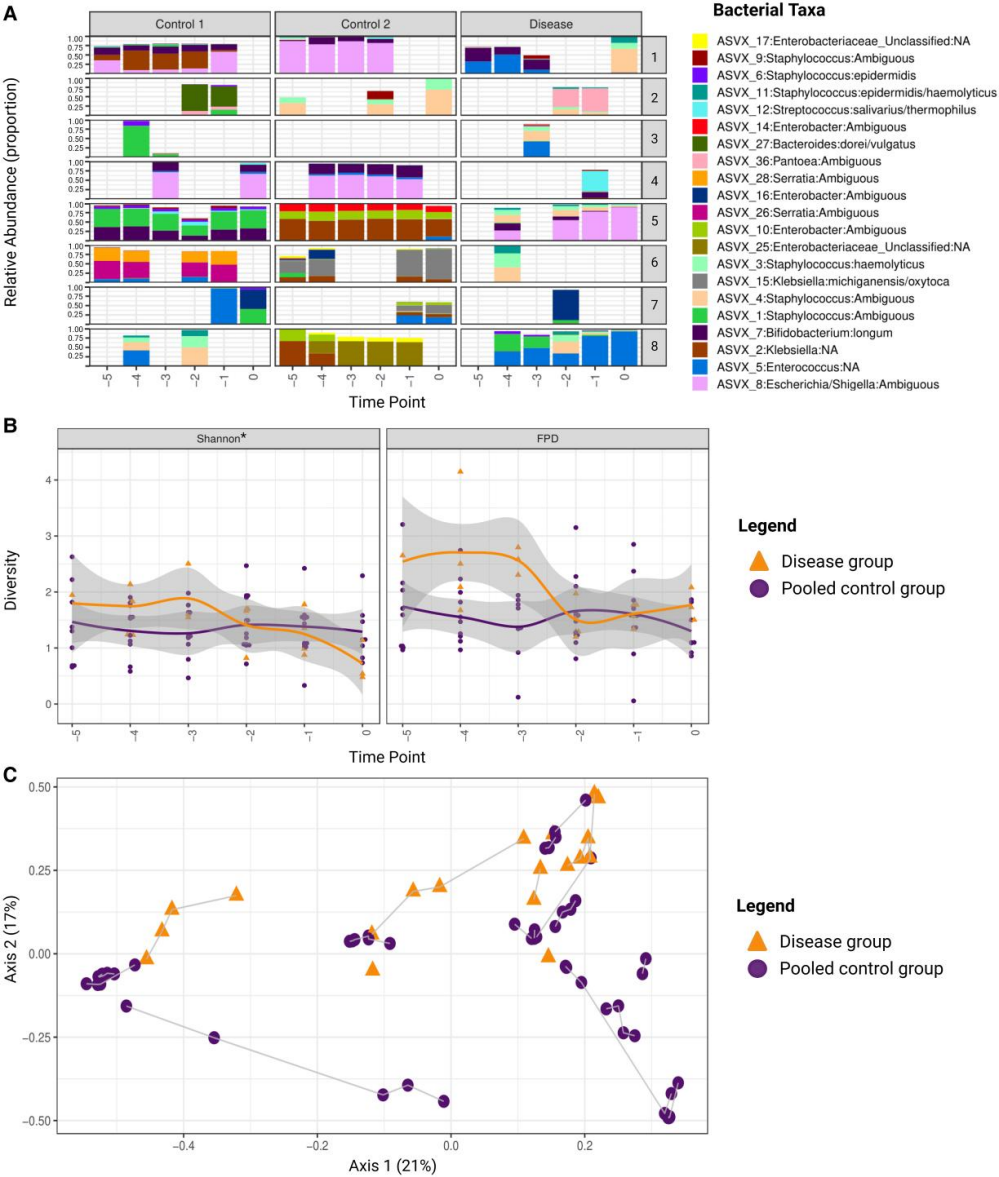
Fecal bacterial microbiota composition in preterm infants with *Candida* late-onset sepsis vs the pooled control group. *A*, Bacterial composition plot shows the relative abundance of amplicon sequence variants in the disease and pooled control groups. On the left y-axis, the relative abundance (proportion) of the bacterial amplicon sequence variants at each time point is displayed with proportions shown at 0, 0.25, 0.50, 0.75, and 1.00. The x-axis shows the time points relative to onset of disease (t = −5 to t = 0). On the right y-axis, the match groups are displayed. Each match group consists of 1 case and 2 nonaffected control infants. Each color represents a specific bacterial species. *B*, Longitudinal course of bacterial α-diversity from 5 days prior to clinical onset (t = −5) until the day of diagnostic workup (t = 0) in infants with *Candida* late-onset sepsis (disease group, orange) vs matched control infants (pooled control group, purple). The y-axis shows the Shannon diversity index (left) and Faith phylogenetic diversity (right). Differences in overall α-diversity across all time points between the groups were assessed via linear mixed model analysis (Satterthwaite method; Δ = −0.2, *P* = .012, for Shannon diversity index; Δ = −0.12, *P* = .112, for Faith phylogenetic diversity). Shaded areas indicate 95% CI. Statistical significance was defined as *P* ≤ .05. *C*, Bacterial β-diversity at the genus level, visualized by principal coordinate analysis based on Bray-Curtis dissimilarity (*P* = .046, *R*^2^ = 10.5%). Each point represents a single representative fecal sample per infant, colored by disease state (orange, disease group; purple, pooled control group). The gray lines connect samples belonging to the same matched group. Due to the sparsity of the data, 1 sample per participant was randomly selected to test differences in β-diversity by permutational multivariate analysis of variance. To account for potential selection bias, the random sample selection was repeated 100 times, and average statistical metrics and *P* values were calculated. Statistical significance was defined as *P* ≤ .05. Created in BioRender. Amsterdamumc, Eminds (2025) https://BioRender.com/9d5rwly

Similar to the fungal analysis, we compared the pooled control group with the disease group to assess the bacterial signatures, as no relevant differences were observed between the control subgroups (Figure 4*A*). The intestinal microbiota was highly variable between individuals in the disease and pooled control groups, reflecting interindividual differences. However, within each participant, the bacterial composition remained relatively stable over time (Figure 4*A*, Supplementary Figure 2B). The predominant bacterial taxa in both groups included *Enterococcus*, *Staphylococcus*, members of the Enterobacteriaceae family (*Klebsiella*, *Escherichia/Shigella*), and *Bifidobacterium*. A significant decrease in α-diversity, as measured by the Shannon index, was observed in the days prior to onset of *Candida* LOS as compared to the pooled control group (Δ = −0.2, *P* = .012), indicating a reduced microbial evenness in the disease group (Figure 4*B*). Bacterial β-diversity, based on the Bray-Curtis dissimilarity index with a single representative fecal sample per infant, revealed significant clustering of samples according to the disease status (*R*^2^ = 10.5%, *P* = .046; Figure 4*C*). Despite this separation at the sequence variant level, no specific genera were consistently associated with *Candida* LOS. Although some genera differed significantly, their low abundance levels suggest that these findings may be spurious (Supplementary Figure 4).

### Fecal Fungal Yield Correlates Positively With *Candida albicans* Prior to Clinical Onset of *Candida* LOS

Next, we evaluated the relationship between fecal microbiota and mycobiota in the disease and the pooled control groups per the Procrustes test, which revealed a significant positive correlation between the overall community structures of the 2 kingdoms (*P* = .001). However, correlation analysis at the species level, based on a single representative fecal sample per infant, did not identify specific associations between the bacterial microbiota and fungal mycobiota. Finally, we investigated whether the increased fungal yield observed in the disease group was associated with the abundance of *C albicans*. A significant positive correlation was observed between the relative abundance of *C albicans* and total fungal yield in infants with *Candida* LOS (ρ = 0.71, adjusted *P* = .005), suggesting a higher absolute abundance of *C albicans* in the preclinical fecal samples of the disease group as compared to the pooled control group.

## DISCUSSION

This study provided novel insights into the intestinal microbial dynamics preceding *Candida* LOS in very preterm infants. Our findings demonstrate increased fecal *C albicans* in infants who developed *Candida* LOS, accompanied by a reduced bacterial amplicon yield and lower α-diversity, up to 5 days prior to clinical onset. While the skin-to-blood route is most often referred to in the disease development of *Candida* LOS [5, 30, 31], our results support a gut-to-blood route of infection, suggesting a paradigm shift in understanding *Candida* LOS.

Building on this hypothesis of gut-associated *Candida* LOS, experimental models have demonstrated that *C albicans* possesses virulence factors that facilitate endothelial and epithelial disruption, enabling translocation across host barriers. Among these, adhesins play a key role in promoting mucosal adhesion and biofilm formation, while hyphal transition and toxin and enzyme secretion are essential for epithelial invasion and immune responses [32–35]. Although these virulence factors are well characterized and the GI tract is recognized as a reservoir for *C albicans*, particularly among adults who are immunocompromised [22 , 23, 36, 37], evidence specifically addressing gut-derived *Candida* LOS in preterm infants is limited [22, 24]. Considering our findings and the inherent ability of *Candida* to colonize and invade the immature intestinal epithelium, we propose that intestinal translocation may represent a clinically relevant route of infection.

Given the intricate interplay between bacterial and fungal communities in the gut [38–40], we also characterized the fecal microbiota prior to *Candida* LOS to explore the potential contributions of bacteria in the disease development. Both the disease and pooled control groups were predominantly colonized by early facultative anaerobic bacteria, such as Lactobacillales, Enterobacteriaceae, *Bifidobacterium*, and *Staphylococcus* [41]. While we did not identify specific bacterial taxa associated with *Candida* LOS development, we observed a reduced bacterial yield and α-diversity prior to onset. Shannon diversity decreased significantly prior to *Candida* LOS, while Faith phylogenetic diversity showed a similar but nonsignificant trend, as expected given their different community structures. Notably, Faith phylogenetic diversity was initially elevated in the disease group before declining, suggesting temporary instability or compositional turnover, potentially involving low-abundance taxa. Overall, these findings suggest a more limited and ultimately less diverse ecologic GI niche, potentially favoring interkingdom competition and fungal overgrowth. *Candida* species are known to interact, support, and compete with bacterial colonizers in the GI tract [40, 42]. The microbiota plays an important role in regulating *Candida* abundance in the gut, as supported by the fact that exposure to empiric antibiotics is a risk factor for candidemia [27]. Our findings suggest that microbiota signatures may promote *C albicans* expansion and translocation across the compromised preterm intestinal barrier [43].

To assess whether the fungal signatures observed prior to *Candida* LOS could be attributed to extensive antibiotic exposure, a known risk factor for intestinal colonization and overgrowth of *C albicans* [25–28], or were truly disease specific, infants in control group 2 were matched to cases based on cumulative antibiotic exposure. In contrast, control group 1 was not matched for antibiotic exposure. Distinct fungal and bacterial signatures persisted in the disease group, while the control groups displayed similar fungal and bacterial profiles despite differences in antibiotic exposure. These findings suggest that *C albicans* overgrowth in the disease group is not solely a consequence of antibiotics but may be a disease-specific feature related to underlying vulnerability or altered microbial ecology. Notably, in addition to frequent GI comorbidities prior to onset of *Candida* LOS, we found that all infants in the disease group were born vaginally, which may contribute to vertical transmission of *Candida* in this population [44]. Future research should clarify to what extent delivery mode in this population contributes to intestinal *C albicans* overgrowth and systemic invasion.

Our study is limited by the small sample size, which reflects the low incidence of *Candida* LOS (0.8%) and limited availability of fecal samples (available for 42% cases). Additionally, our study was limited to the prospective collection of fecal samples. While we have access to the culture results on a species level, blood-derived isolates were unavailable for whole genome sequencing. Increased intestinal *Candida* colonization may coexist with skin colonization, keeping a skin-to-blood route plausible. Future research should incorporate strain tracking by the direct comparison of the genetic sequence of blood and/or CSF *Candida* isolates with those from fecal samples. Moreover, the majority of fungal LOS cases were caused by *C albicans*, which limits the generalizability of our findings to other species. In the single *C tropicalis* case, intestinal colonization was restricted to this species, but no firm conclusion can be drawn from this single case. Importantly, fluconazole prophylaxis is not routinely administered in the Netherlands and Belgium, constraining external validity to settings where prophylactic use is standard practice. Last, we assessed the fungal and bacterial yields by quantifying total amplicons normalized to the DNA stool concentration. While informative, this approach has limitations, including amplification bias and restricted taxonomic resolution. To account for potential DNA isolation or PCR biases, we determined the ITS/16S amplicon yield ratio as a relative measure of fungal vs bacterial contribution, which was significantly increased in the affected infants. Future studies should incorporate fungal culturing from fresh samples or spiking DNA isolates with defined quantities of reference fungi and bacteria to complement sequencing-based approaches, while considering the inherent limitations of these techniques.

In conclusion, we observed increased fecal *C albicans* preceding *Candida* LOS in very preterm infants, supporting the hypothesis that the preterm gut may serve as a source of infection for fungal pathogens. These findings challenge the traditional view of skin-to-blood transmission as the primary route of infection and suggest a potential paradigm shift in our understanding on the etiology of *Candida* LOS. Preventive strategies have traditionally focused on skin integrity management to reduce *Candida-*associated morbidity and mortality. However, our results indicate that gut colonization plays a critical role, underscoring opportunities for improved risk stratification and development of novel targeted interventions to prevent onset and progression of *Candida* LOS in high-risk infants.

## Supplementary Material

jiaf524_Supplementary_Data

## Supplementary Data

Supplementary materials are available at *The Journal of Infectious Diseases* online (http://jid.oxfordjournals.org/). Supplementary materials consist of data provided by the author that are published to benefit the reader. The posted materials are not copyedited. The contents of all supplementary data are the sole responsibility of the authors. Questions or messages regarding errors should be addressed to the author.
