# Supplementary material for: The Gut as a Source of Infection for Fungal Pathogens: Increased Fecal Candida albicans Precedes Onset of Candida Late-Onset Sepsis in Very Preterm Infants
Source: J Infect Dis. 2025 Oct 10;233(2):e570–81. doi: 10.1093/infdis/jiaf524 (PMC13017767; doi:10.1093/infdis/jiaf524)
Supplement: jiaf524_Supplementary_Data [file jiaf524_supplementary_data.docx]

**Supplementary Materials**

Corresponding manuscript title: *The gut as a source of infection for fungal pathogens: increased fecal Candida albicans precedes onset of Candida late-onset sepsis in very preterm infants*

Authors:

Rimke R. de Kroon*, Irini A.M. Kreulen*, Mark Davids, Isabelle A.M. van Thiel, Iris Admiraal, Xanthe Verdoes, Mirjam M. van Weissenbruch, Hendrik Niemarkt, Wouter J. de Jonge, Tim de Meij, on behalf of the generation P study group

*Contributed equally to the project

**Appendix A**

Microbial DNA isolation from neonatal faeces

Microbial DNA was isolated from fecal samples using the PSP Spin Stool DNA Plus Kit (Invitek Molecular/Isogen Life Science, De Meern, the Netherlands) with minor adjustments to the protocol, as described below. The frozen faecal samples were subsequently homogenized in Stool DNA Stabilizer buffer (Isogen) by using glass based lysing Matrix beads (MilliPore biochemicals Lysing Matrix type E) and Precellys 24 Touch Homogenizer (3 x 30 sec, 6.5 ms^-2^, Bertin Technologies). Thereafter, samples were heated at 95℃ for 15 minutes followed by cooling on ice. The fecal derived supernatants were transferred to the PSP InviAdsorb tubes and manufacturer’s kit protocol was followed from this step onwards. Negative procedural control samples were included and handled the same as the faecal samples.

Sequencing fungal and bacterial profiles in neonatal fecal samples

ITS1 amplicon and 16S rRNA gene sequencing was performed by the Microbiota Centre Amsterdam (MiCA). The fungal composition was determined by ITS1 amplicon sequence analysis. PCR-generated amplicon libraries were obtained from 100 ng fecal DNA using the ITS1 primer set (**Table S5**) containing an overhang for the Illumina Nextera platform and Phusion high-fidelity DNA polymerase (Thermo Fisher Scientific, Waltham, MA, USA). A duplicate reaction in 20 µl was performed with the following thermocycling conditions: an initial denaturation step at 98°C for 1 min followed by 35 cycles of denaturation (20 s), annealing (20 s at 58°C), and extension (60 s at 72°C), and a final extension step (5 min at 72°C). The duplicates were pooled to a final volume of 40 µl. A second amplification step was used to introduce multiplex indices and the Illumina sequencing adapters using the Kapa polymerase system. The reaction was performed in 20 µl using the following conditions: initial denaturation (3 min at 95°C), followed by 24 cycles of denaturation (20 s at 98°C), annealing (20 s at 60°C), and extension (60 s at 72°C), and a final extension step (5 min at 72°C).

Additionally, 16S rRNA gene amplicon sequencing was performed, as previously described (45, 46). In brief, following DNA Isolation, 16S rRNA gene amplicons containing barcoded V3-V4 region of the 16S genes were amplified with Index primers with an adapted PCR. The forward Indexprimer (Illumina Adapter,Index,pad,link,16Sf) and reversed Indexprimer (Illumina Adapter,Index,pad,link,16Sr) were used. 20 ng of template DNA was used in the PCR reaction with the following conditions: an initial denaturation step (10 s at 98°C), followed by 25 cycles of denaturation (10 s at 98°C), annealing (55 s at 20°C), and extension (90 s at 72°C), and a final extension step (10 min at 72°C).

Both fungal and bacterial PCR products, as described above, were purified using AMPure XP beads (Beckman Coulter, Brea, CA, USA). The total fecal DNA concentration per samples as well as amplicon DNA concentration (16S- and ITS PCR) was measured using the Qubit fluorometric quantitation method (Thermo Fisher Scientific, Waltham, MA, USA), and DNA quality was determined using the Agilent Bioanalyzer DNA-1000 chip, after which the purified products were equimolarly pooled. The libraries were sequenced in a paired-end run with 250 cycles on an Illumina MiSeq platform (GATCBiotech, Constance, Germany) using V3 chemistry.

Preprocessing of sequencing data

Preprocessing of the data included filtering the Amplified Sequence Variants (ASVs) on kingdoms and removing non-fungal or bacterial kingdoms. The reads were merged and trimmed using USEARCH (47). Quality controls were implemented by excluding reads via the Illumina chastity filter, with high error scores (i.e., > 2) or shorter than 380 base pairs. Amplified Sequence Variants (ASVs) were inferred from each sample individually, with a minimum abundance of 4 reads per sample (48). The collective ASV set was used to establish the abundances of unfiltered reads. The bacterial taxonomy was assigned using the IDTaxa (41) and SILVA 16S ribosomal database V132 (49). The ASVs identified by the prediction model were subjected to BLAST (Basic Local Alignment Search Tool, NIH (50)).

**Supplementary Tables**

**Supplementary Table 1A:** Overview of infants and sample distribution for ITS1 amplicon sequencing.

|  | **Infants enrolled/ drop out** | **Total number of infants analyzed** | **Total fecal samples/drop out** | **Fecal samples analyzed** |
| --- | --- | --- | --- | --- |
| **Disease** | 8/0 | 8 | 22/4 | 18 |
| **Pooled control** | 16/0 | 14 | 56/14 | 42 |
| - Control 1 | 8/0 | 8 | 25/3 | 22 |
| - Control 2 | 8/2 | 6 | 31/11 | 20 |

**Supplementary Table 1B:** Overview of infants and sample distribution for 16S rRNA sequencing.

|  | **Infants enrolled/ drop out** | **Total number of infants analyzed** | **Total fecal samples/drop out** | **Fecal samples analyzed** |
| --- | --- | --- | --- | --- |
| **Disease** | 8/0 | 8 | 22/3 | 19 |
| **Pooled control** | 16/2 | 14 | 56/7 | 49 |
| - Control 1 | 8/0 | 8 | 25/0 | 25 |
| - Control 2 | 8/2 | 6 | 31/7 | 24 |

**Supplemental Table 2:** Baseline characteristics of preterm infants with *Candida* LOS (n=19) and the subgroup of *Candida* LOS cases (n=8), which were included in ITS1 amplicon and 16S rRNA gene analysis (disease group, n=8).

|  | **Total cohort (n=19)** | **Disease group (n=8)** | **p-value** |
| --- | --- | --- | --- |
| **Demographic characteristics** | | | |
| Gestational age, median (weeks+days), IQR (days) | 25+2 (14) | 24+5 (13) | 0.979 |
| Birth weight, median, IQR (grams) | 745 (135) | 742 (279) | 0.815 |
| Biological sex, female (n[%]) | 7 [37] | 3 [38] | 0.340 |
| Singleton (n[%]) | 13 [68] | 5 [63] | 0.974 |
| Apgar score at 5 min, median, IQR | 7 (3) | 8 (4) | 0.979 |
| Modus partus, vaginal delivery (n[%]) | 17 [89] | 8 [100] | 0.766 |
| Maternal antenatal corticosteroids (n[%]) -No treatment  -Incomplete  -Complete | 5 [26]  7 [44]  7 [44] | 2 [25]  3 [37.5]  3 [37.5] | 0.997 |
| Mortality within NICU admittance (n[%]) | 6 [32] | 1 {12.5) | 0.302 |
| **Medication practices** | | | |
| Surfactant treatment within 72 hours (n[%]) -Multiple dosages  -1 dosage  -No treatment | 7 [37]  3 [16]  9 [47] | 4 [50]  0 [0]  4 [50] | 0.470 |
| Ratio cumulative iv antimicrobials in first 28 days of life, median (IQR) | 0.83 (0.28) | 0.78 (0.26) | 0.658 |
| Cumulative iv antimicrobials at t=-1, median, IQR (days) | 6 (6) | 11 (11) | 0.283 |
| Exposure to iv antimicrobials prior to t=0 (n[%]) | 19 [100] | 8 [100] | n.a. |
| Exposure to probiotics prior to t = 0 (n[%]) | 4 [21] | 3 [37.5] | 0.373 |
| **Average feeding practices** | | | |
| Reached FEF in first month of life, yes (n[%])^1^ | 12 [64] | 6 [75] | 0.551 |
| Postnatal age at first day of FEF, median, IQR (days) | 14 (14) | 13 (12) | 0.892 |
| Feeding type at first day of FEF: exclusively human milk (n[%]) | 12 [100] | 6 [100] | n.a. |
| Received formula feeding in first month of life (n[%])^2^ | 5 [26] | 2 [25] | 0.751 |
| Reached FEF at t = 0, yes (n[%]) | 5 [26] | 3 [38] | 0.561 |
| ^1^7 infants in the total cohort did not reach enteral feeding within the first month of life or died prior to reaching full enteral feeding; 2 of these infants were also included in the study group. ^2^For 3 infants, there was no data available on formula feeding due to admission prior to electronic patient files. *Abbreviations: FEF = full enteral feeding; iv = intravenously; NICU = neonatal intensive care unit. All p-values* ***≤****0.05 were considered significant.* | | | |

**Supplementary Table 3:** Detailed clinical characteristics of preterm infants with *Candida* LOS included in the ITS1 amplicon and 16S rRNA gene analysis (n=8)

| **Clinical characteristics** | **Disease group (n=8)** |
| --- | --- |
| Day of life clinical suspicion of invasive fungal infection, median, IQR. | 19 (14) |
| Highest CRP measured at time of clinical suspicion, median, IQR. | 49 (103) |
| Pathogen cultured from:  - Only from blood culture (n[%]) - Only from CSF culture (n[%]) - Blood and CSF culture (n[%]) | 5 [62.5]  1 [12.5]  2 [25.0] |
| Cultured pathogen:  -*Candida albicans* (n[%]) -*Candida tropicalis* (n[%]) | 7 [87.5]  1 [12.5] |
| Exposure to invasive medical device (including invasive ventilation, central venous catheter, intravenous catheter) in 48 hours prior to clinical onset of disease, yes [n%]) | 8 [100] |
| Involved organs:  - Liver (n[%])  - Kidneys (n[%])  - Eyes (n[%])  - Cerebrum (n[%])  - Joints/bones (n[%]) | 2 [25]  1 [13]  1 [13]  3 [38]  1 [13] |
| Absence of concurrent comorbidities, yes (n[%]) | 2 [11] |
| Fungal pathogen cultured from urine culture prior to clinical suspicion of *Candida* LOS (n[%]) | 2 [25] |
| Clinical suspicion of fungal skin infection that required treatment with miconazole prior to clinical suspicion of *Candida* LOS (n[%]) | 5 [63] |
| Comorbidities (including clinical sepsis, blood culture-proven EOS or LOS, NEC stage 2A or higher, SIP, or combination) prior to invasive fungal infection, yes (n[%]) | 5 [63] |
| *Abbreviations: CRP = C-reactive protein, CSF = cerebrospinal fluid; EOS = early-onset sepsis; SIP = spontaneous intestinal perforation; LOS = late-onset sepsis, NEC = necrotizing enterocolitis.* | |

**Supplementary Table 4: Raw ITS and 16S data per sample.** This table displays the DNA yield (ng/μL), 16S amplicon yield (ng/μL), ITS amplicon yield (ng/μL), ITS/16S amplicon yield ratio, and 16S and ITS read counts per sample. For each sample, input fecal DNA was normalized 20 ng for 16S, 100 ng for ITS PCR reactions to minimize variation in DNA extraction concentrations. The ITS/16S amplicon yield ratio was calculated to account for PCR bias from unclassified, non-target DNA fractions. Metadata for each sample includes patient ID, timepoint, and group (disease or control).

| **Sample ID** | **Patient ID** | **Time point** | **Group** | **DNA yield (ng/μL)** | **16S amplicon yield (ng/μL)** | **ITS amplicon yield (ng/μL)** | **ITS/16S amplicon yield ratio** | **16S reads** | **ITS reads** |
| --- | --- | --- | --- | --- | --- | --- | --- | --- | --- |
| 1 | 1 | 0 | Control | 74.93 | 17.82 | 3.84 | 0.22 | 20049 | 98686 |
| 2 | 1 | -3 | Control | 89.23 | 11.68 | 4.44 | 0.38 | 30834 | 65249 |
| 3 | 2 | 0 | Disease | 47.24 | 7.00 | 27.60 | 3.94 | 25055 | 153934 |
| 4 | 2 | -5 | Disease | 23.13 | 12.95 | 22.40 | 1.73 | 13208 | 44645 |
| 5 | 2 | -4 | Disease | 80.90 | 13.37 | 16.00 | 1.20 | 16281 | 76213 |
| 6 | 2 | -3 | Disease | 66.03 | 14.84 | 14.60 | 0.98 | 12668 | 181706 |
| 7 | 3 | -5 | Control | 174.25 | 13.56 | 2.29 | 0.17 | 15692 | 106506 |
| 8 | 3 | -1 | Control | 182.10 | 12.22 | 23.60 | 1.93 | 21208 | 28279 |
| 9 | 3 | -4 | Control | 414.36 | 19.77 | 2.03 | 0.10 | 18410 | 22588 |
| 10 | 3 | -3 | Control | 127.30 | 20.15 | 0.89 | 0.04 | 18145 | 20572 |
| 11 | 3 | -2 | Control | 130.48 | 13.55 | 4.36 | 0.32 | 22406 | 88446 |
| 12 | 4 | -1 | Disease | 34.48 | 9.70 | 14.40 | 1.48 | 25219 | 124032 |
| 13 | 5 | 0 | Control | 4.13 | 16.53 | 0.76 | 0.05 | 21208 | 16396 |
| 14 | 5 | -2 | Control | 6.01 | 14.64 | 3.40 | 0.23 | 19689 | 13861 |
| 15 | 5 | -5 | Control | 22.08 | 15.84 | 1.56 | 0.10 | 20352 | 27007 |
| 16 | 6 | -1 | Control | 20.64 | 13.23 | 22.10 | 1.67 | 21495 | 30343 |
| 17 | 6 | 0 | Control | 60.41 | 15.21 | 24.30 | 1.60 | 22724 | 68574 |
| 18 | 7 | -2 | Control | 282.04 | 14.16 | 2.92 | 0.21 | 34072 | 26921 |
| 19 | 7 | -1 | Control | 357.94 | 19.33 | 3.10 | 0.16 | 31995 | 9275 |
| 20 | 8 | 0 | Control | 68.60 | 14.61 | 1.70 | 0.12 | 16108 | 55130 |
| 21 | 8 | -1 | Control | 513.27 | 17.76 | 1.55 | 0.09 | 17604 | 399 |
| 22 | 9 | -2 | Disease | 15.52 | 12.76 | 27.60 | 2.16 | 26378 | 136991 |
| 23 | 9 | -1 | Disease | 15.12 | 13.00 | 14.56 | 1.12 | 28582 | 260228 |
| 24 | 10 | -2 | Disease | 71.50 | 3.28 | 20.80 | 6.34 | 32164 | 45233 |
| 25 | 11 | -5 | Control | 31.37 | 16.40 | 1.46 | 0.09 | 12878 | 5684 |
| 26 | 11 | -4 | Control | 72.81 | 10.94 | 0.99 | 0.09 | 18311 | 6308 |
| 27 | 11 | 0 | Control | 45.64 | 14.18 | 0.95 | 0.07 | 16688 | 5921 |
| 28 | 11 | -1 | Control | 100.19 | 13.36 | 0.82 | 0.06 | 19813 | 39935 |
| 29 | 12 | -1 | Control | 21.18 | 17.35 | 3.33 | 0.19 | 23308 | 121684 |
| 30 | 12 | -4 | Control | 21.16 | 18.27 | 0.77 | 0.04 | 18136 | 14062 |
| 31 | 12 | -2 | Control | 22.77 | 15.43 | 7.50 | 0.49 | 16929 | 320900 |
| 32 | 12 | -5 | Control | 27.51 | 16.51 | 2.06 | 0.12 | 23902 | 125472 |
| 33 | 13 | -4 | Disease | 124.22 | 1.17 | 34.50 | 29.49 | 22563 | 129815 |
| 34 | 14 | -5 | Control | 249.98 | 12.33 | 15.00 | 1.22 | 26755 | 138778 |
| 35 | 14 | -4 | Control | 341.31 | 14.82 | 24.00 | 1.62 | 23600 | 19836 |
| 36 | 14 | -3 | Control | 178.28 | 10.42 | 15.00 | 1.44 | 27959 | 52025 |
| 37 | 14 | -2 | Control | 171.60 | 16.30 | 2.27 | 0.14 | 20619 | 31528 |
| 38 | 15 | -3 | Control | 46.45 | 11.66 | 1.07 | 0.09 | 18381 | 21034 |
| 39 | 15 | -5 | Control | 53.95 | 18.00 | 1.82 | 0.10 | 19910 | 40170 |
| 40 | 15 | -4 | Control | 15.23 | 18.27 | 0.74 | 0.04 | 22898 | 9782 |
| 41 | 15 | -2 | Control | 42.71 | 14.46 | 1.00 | 0.07 | 20840 | 31030 |
| 42 | 15 | -1 | Control | 25.81 | 15.63 | n.a. | n.a. | 15478 | n.a. |
| 43 | 15 | 0 | Control | 12.26 | 17.35 | 1.09 | 0.06 | 17296 | 6044 |
| 44 | 16 | -4 | Disease | 46.74 | 9.39 | 0.52 | 0.05 | 18213 | 1753 |
| 45 | 16 | -2 | Disease | 61.87 | 11.20 | 1.14 | 0.10 | 22753 | 17632 |
| 46 | 16 | -1 | Disease | 244.88 | 13.58 | 13.40 | 0.99 | 22793 | 865 |
| 47 | 16 | 0 | Disease | 512.29 | 16.43 | 8.24 | 0.50 | 29774 | 60115 |
| 48 | 17 | -5 | Control | 57.33 | 14.35 | 2.55 | 0.18 | 20133 | 5812 |
| 49 | 17 | -4 | Control | 423.31 | 14.10 | 1.45 | 0.10 | 15205 | 2665 |
| 50 | 17 | -3 | Control | 74.59 | 13.68 | 2.49 | 0.18 | 23001 | 1005 |
| 51 | 17 | -1 | Control | 130.62 | 12.18 | 0.98 | 0.08 | 27008 | 4005 |
| 52 | 17 | -2 | Control | 1269.98 | 12.10 | 1.67 | 0.14 | 29213 | 68839 |
| 53 | 18 | -4 | Control | 42.32 | 14.95 | n.a. | n.a. | 22702 | n.a. |
| 54 | 18 | -3 | Control | 15.06 | 5.02 | 2.60 | 0.52 | 33652 | 22719 |
| 55 | 19 | -2 | Control | 7.50 | 4.27 | 0.59 | 0.14 | 28417 | 2985 |
| 56 | 19 | -4 | Control | 33.56 | 16.14 | 1.43 | 0.09 | 16511 | 102904 |
| 57 | 20 | -3 | Disease | 25.74 | 5.66 | 10.40 | 1.84 | 22024 | 356274 |
| 58 | 20 | -4 | Disease | 72.11 | 0.05 | 24.00 | 480.00 | 2310 | 167637 |
| 59 | 20 | -5 | Disease | 127.74 | 0.03 | 27.80 | 926.67 | 367 | 184426 |
| 60 | 21 | -2 | Disease | 9.85 | 12.66 | 2.65 | 0.21 | 14102 | 2897 |
| 61 | 22 | -3 | Disease | 39.28 | 14.35 | 16.70 | 1.16 | 13496 | 38363 |
| 62 | 22 | -1 | Disease | 10.07 | 8.98 | 19.56 | 2.18 | 16183 | 61578 |
| 63 | 22 | 0 | Disease | 42.86 | 10.06 | 19.70 | 1.96 | 15777 | 44812 |
| 64 | 22 | -5 | Disease | 174.90 | 0.10 | n.a. | n.a. | 3464 | n.a. |
| 65 | 22 | -4 | Disease | 2.47 | 0.50 | 4.56 | 9.12 | 31361 | 84728 |
| 66 | 23 | -3 | Control | 429.44 | 14.23 | 19.70 | 1.38 | 16998 | 91166 |
| 67 | 23 | -4 | Control | 3.45 | 6.29 | 16.00 | 2.54 | 18908 | 100179 |
| 68 | 23 | -2 | Control | 438.06 | 15.30 | 6.70 | 0.44 | 16165 | 270108 |
| 69 | 23 | -1 | Control | 321.69 | 9.71 | 18.00 | 1.85 | 15035 | 242589 |
| 70 | 23 | -5 | Control | 706.11 | 11.32 | 5.68 | 0.50 | 16918 | 147720 |
| 71 | 23 | 0 | Control | 206.40 | 15.43 | 12.48 | 0.81 | 14216 | 56571 |
| 72 | 24 | -4 | Control | 343.00 | 17.01 | 0.60 | 0.04 | 21180 | 648 |
| 73 | 24 | -3 | Control | 352.00 | 16.04 | 0.72 | 0.04 | 18117 | 271 |
| 74 | 24 | -2 | Control | 251.00 | 16.83 | 1.08 | 0.06 | 18236 | 300 |
| 75 | 24 | -1 | Control | 451.00 | 12.11 | 0.74 | 0.06 | 23941 | 97 |

**Supplementary Table 5:** ITS1 primer set

| **Primer** | **Sequence** |
| --- | --- |
| Forward primer | TCGTGCGGCAGCGTCAGATGTGTATAAGAGACAGCCTTGGTCTATTTAGAGGAAGTAA |
| Reverse primer | GTCTCGTGGGCTCGGAGATGTGTATAAGAGACAGGCGGTTCTTCATCGATGC |

**Supplementary Figures**


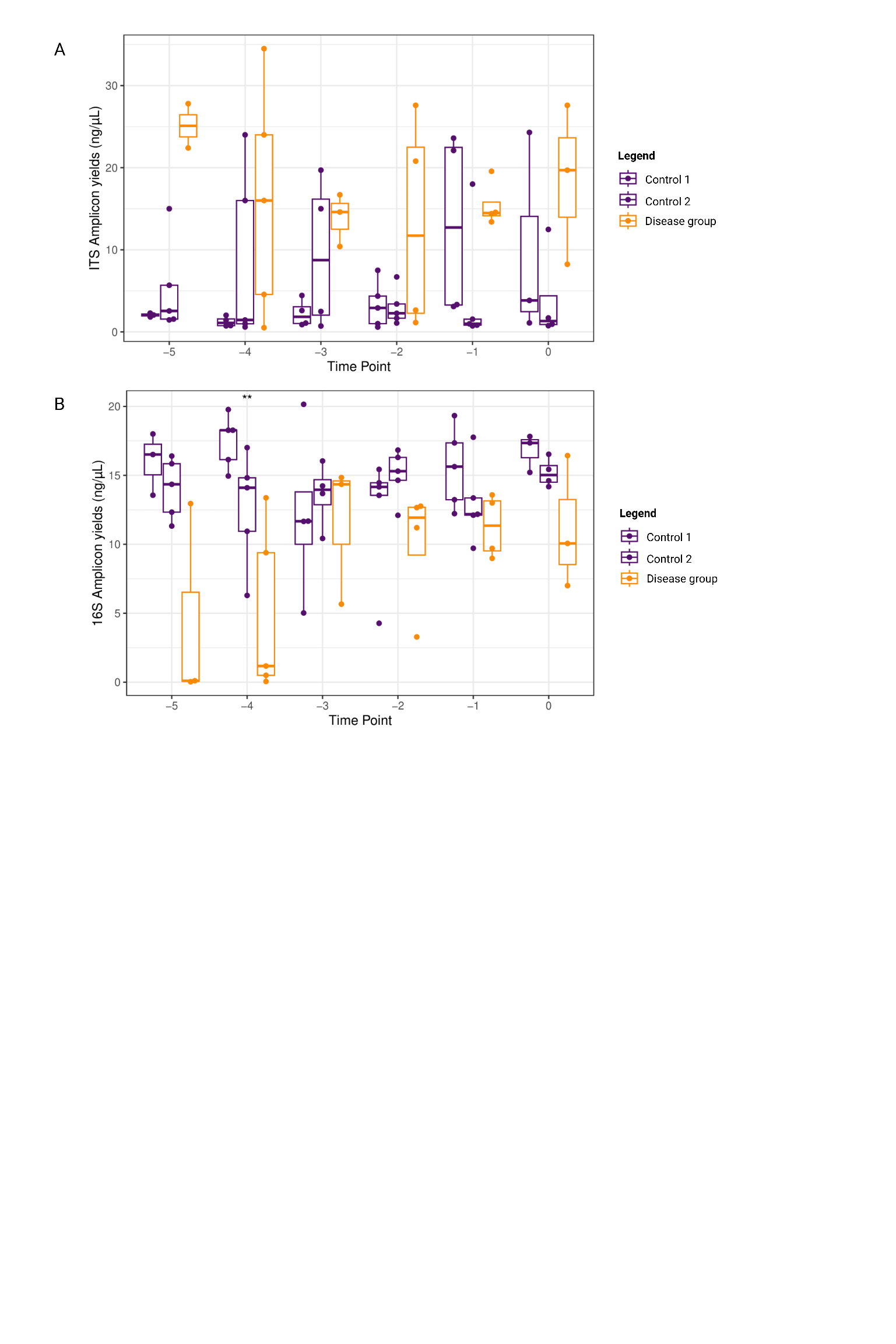


**Supplemental Figure 1: Higher fecal fungal yields and lower bacterial yields in preterm infants with *Candida* late-onset sepsis compared to control group 1 and control group 2.** Fecal fungal amplicon yields (ng/μl) (**S1A**) and bacterial amplicon yields (ng/μl) (**S1B**) determined from five days before clinical diagnosis (t=-5) up to the day of diagnosis (t=0) in infants with *Candida* LOS (disease group, n=8 infants; orange) versus control group 1 (n=8) and control group 2 (n=8; purple). Each infant in the disease group (n=8) was matched to two non-affected control infants (n=16); infants in control group 1 (n=8) were matched based on gestational age (GA) (±5 days), postnatal age (PNA) at diagnostic work-up (t=0) of the matched infant in the disease group, and the hospital site; infants in control group 2 (n=8) were matched based on GA (±5 days), PNA at t=0, and cumulative days of antibiotic administration at t=-1 (±7 days). Differences in overall fungal and bacterial yields between groups were assessed using a Linear Mixed Model analysis (Satterthwaite’s method). Statistical significance was defined as a p-value ≤0.05 (* = ≤0.05, ** = ≤0.001). *Abbreviations: GA = gestational age; LOS = late-onset sepsis; PNA = postnatal age.* Created in BioRender. Amsterdamumc, Eminds (2025) https://BioRender.com/fqib3yg


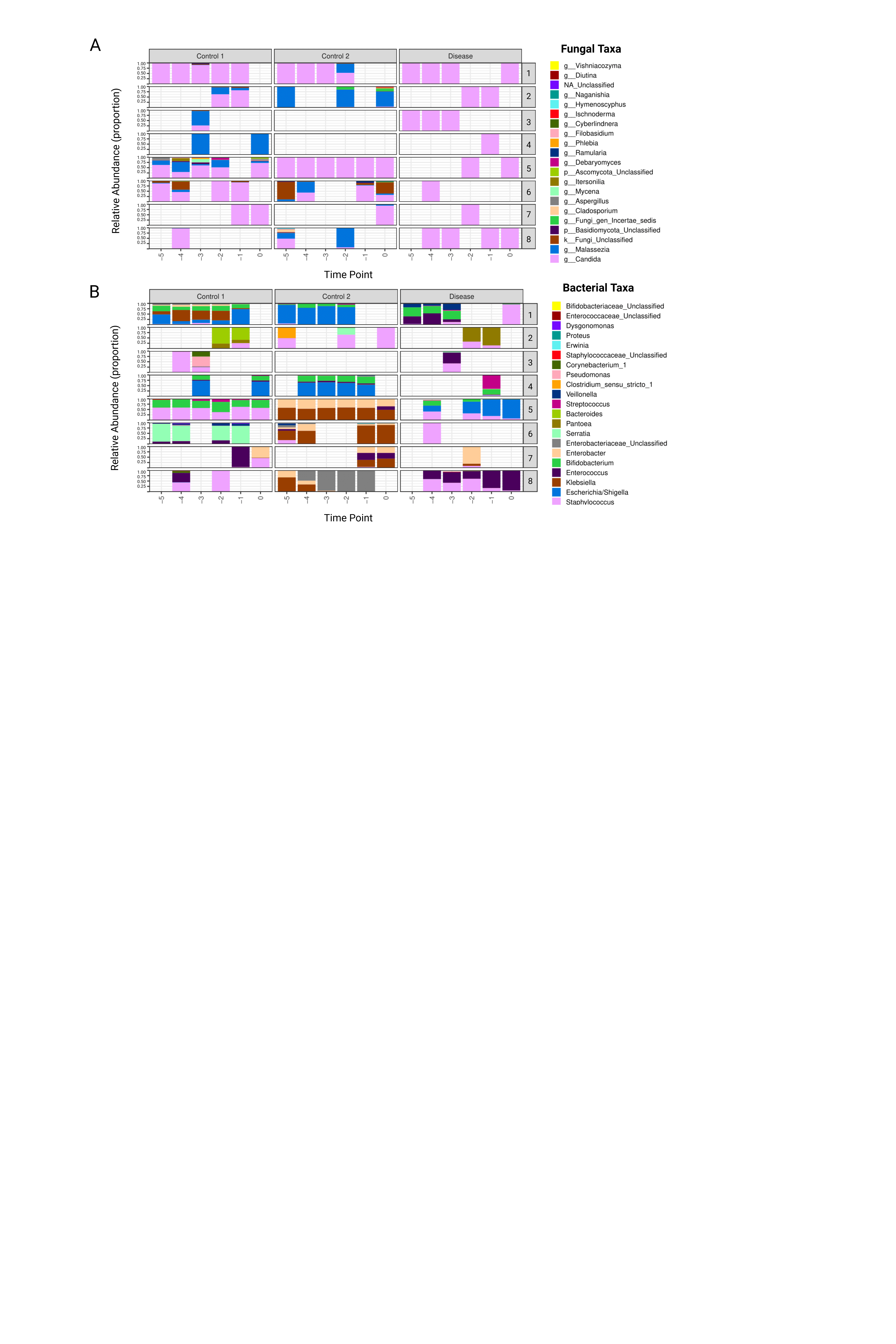


**Supplemental Figure 2:** **Fecal bacterial microbiota and fungal mycobiota composition in preterm infants with *Candida* late-onset sepsis versus the pooled control group.** Bacterial composition plot (**S2A**) and fungal compositional plot (**S2B**) showing the relative abundance (RA) on genus level in the disease and the pooled control group. On the left Y-axis the RA (proportion) of the bacterial ASVs at each time point is displayed with proportions shown at 0, 0.25, 0.50, 0.75 and 1.00. The X-axis shows the timepoints relative to onset of disease (t=-5 to t=0). On the right Y-axis, the match groups are displayed. Each match group consists of one case and two non-affected control infants. Each color represents a specific bacterial genus (**S2A**) or fungal genus (**S2B**). *Abbreviations: ASV = Amplicon Sequence Variants, RA = relative abundance.* Created in BioRender. Amsterdamumc, Eminds (2025) https://BioRender.com/w5o4e4k


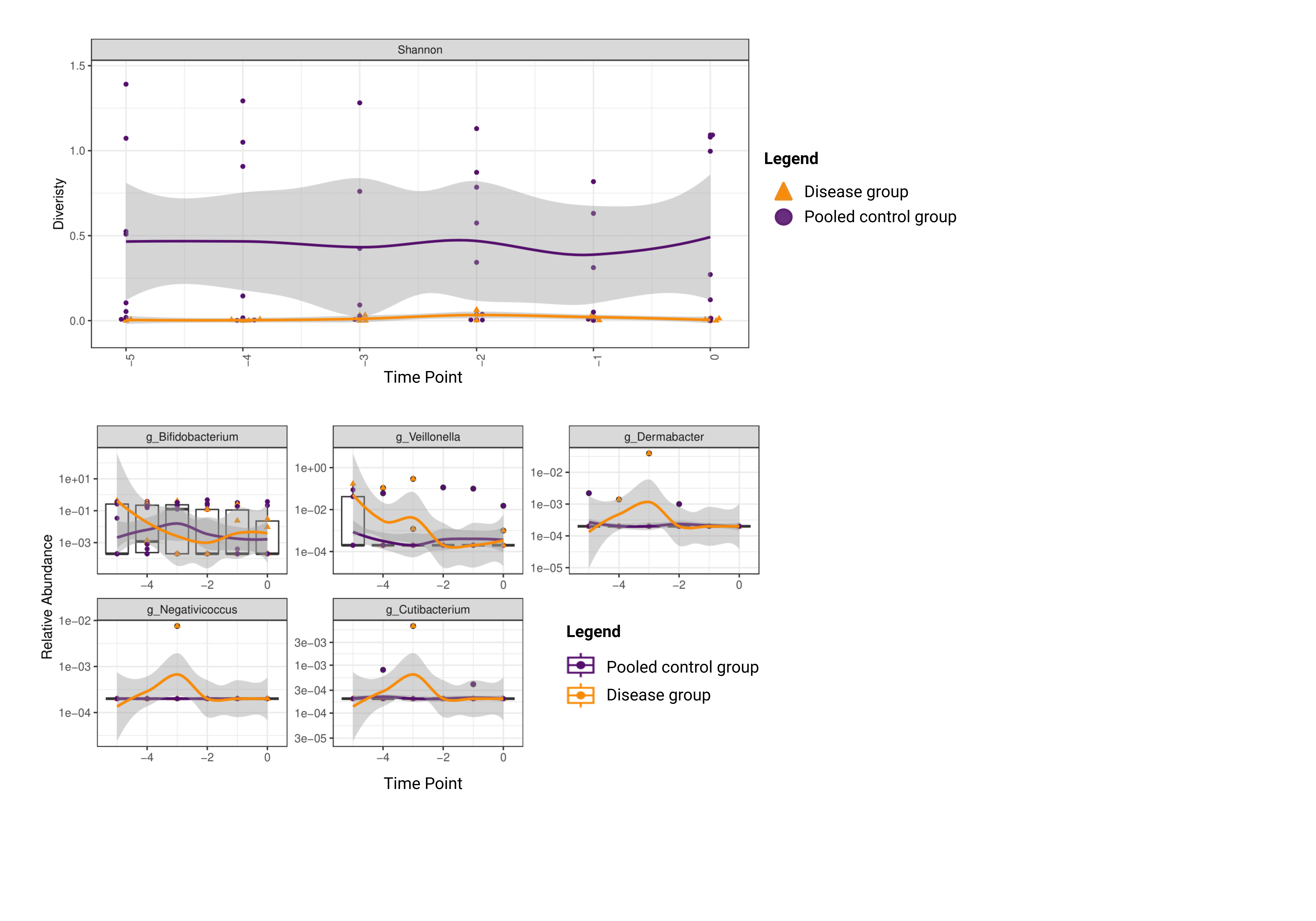


**Supplemental Figure 3**: **Fungal α-diversity in disease group and pooled control group.** Longitudinal course of fungal α-diversity on day of diagnostic work-up and in five days prior to clinical onset of *Candida* LOS in disease group (orange) versus the pooled control group (purple). On the Y-axis the Shannon diversity index is displayed. On the X-axis the time (days) is displayed. Linear Mixed Model analysis (Satterthwaite’s method) was performed to assess the overall α-diversity, considering all timepoints, between the disease and the control group. A p-value of ≤0.05 was considered significant. Created in BioRender. Amsterdamumc, Eminds (2025) https://BioRender.com/xh47u5k


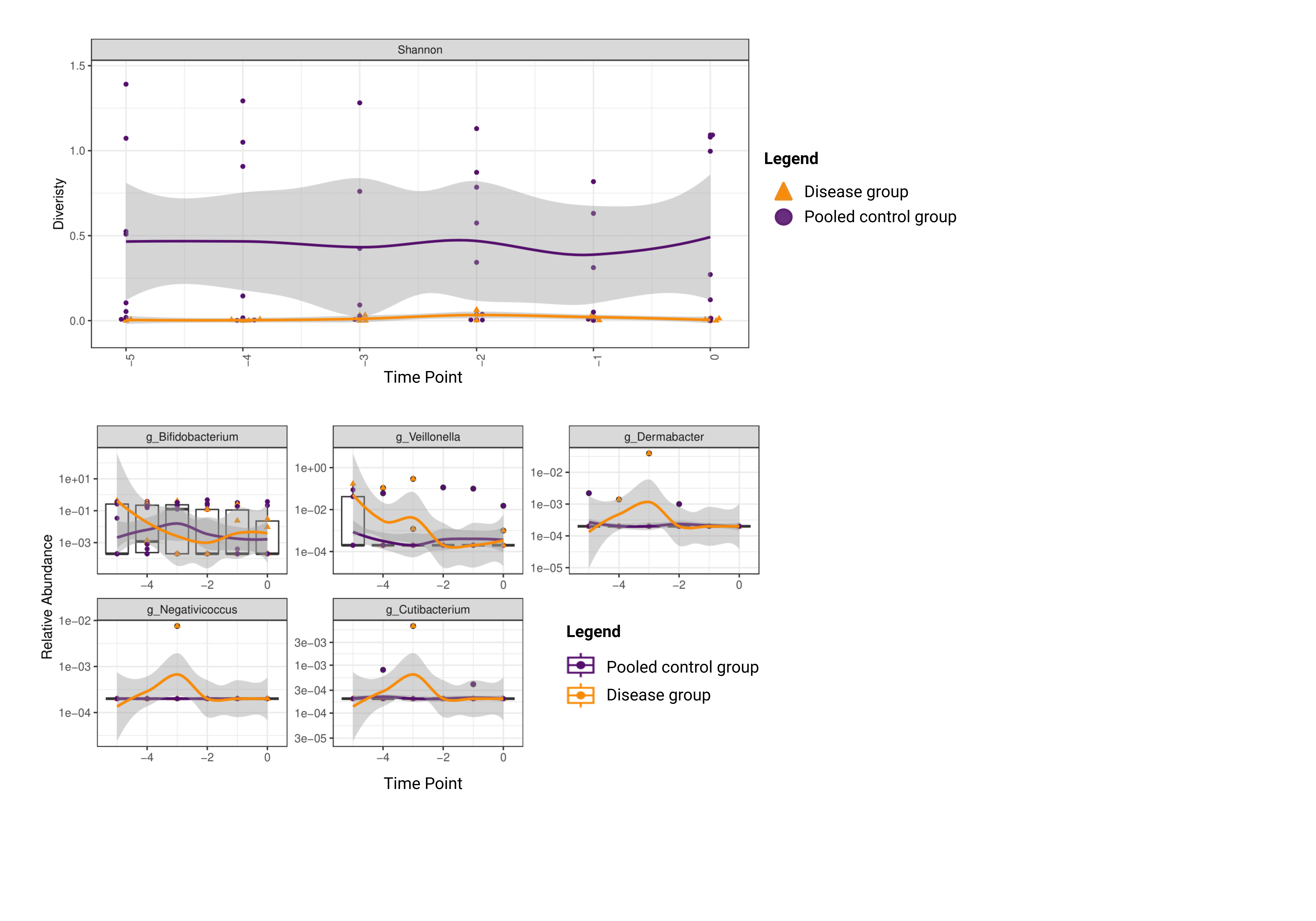


**Supplemental Figure 4**: **Taxa dynamics of *Bifidobacterium, Veillonella, Dermabacter, Negativicoccus*, and *Cutibacterium.*** Taxa dynamics of the bacterial genera (*Bifidobacterium, Veillonella, Dermabacter, Negativicoccus*, and *Cutibacterium,* respectively) on day of diagnostic work-up and five days prior to clinical onset of *Candida* LOS in disease group (orange) versus the pooled control group (purple). On the Y-axis the RA (proportion) of respective bacterial genus is displayed. On the X-axis the time (days) is displayed. Linear Mixed Model analysis (Satterthwaite’s method) was performed to assess the course of the RA in the disease and the pooled control group. A p-value of ≤0.05 was considered significant. While the effects of these bacterial genera were reported as significant, visualization displays that the taxa abundance is low and results are likely spurious. Created in BioRender. Amsterdamumc, Eminds (2025) https://BioRender.com/a4oi75k
